# The effect of yoga training on postpartum prolactin and oxytocin levels in primipara women

**DOI:** 10.25122/jml-2023-0390

**Published:** 2024-02

**Authors:** Reni Yuli Astutik, Noor Pramono, Hardhono Susanto, Martha Irene Kartasurya

**Affiliations:** 1 Doctoral Study Program of Medical and Health Science, Diponegoro University, Semarang, Indonesia; 2 Public Health Nutrition Department, Faculty of Public Health, Diponegoro University, Semarang, Indonesia

**Keywords:** yoga, prolactin, oxytocin, primipara, postpartum

## Abstract

Lactation relies on the secretion of two key hormones, prolactin and oxytocin. Studies have shown that yoga in the postpartum period can stimulate feelings of comfort and relaxation, which increases oxytocin production. The aim of this study was to evaluate the effect of yoga training on postpartum prolactin and oxytocin levels in a group of primipara women. This quasi-experimental study included 60 healthy primigravida, primipara women in their third trimester who attended antepartum and postpartum care at four primary healthcare centers in Kediri Regency. The participants were randomly allocated to an intervention group (*n* = 30) and a control (*n* = 30) group. The intervention group received health education and participated at eight yoga sessions with a duration of 60 min, from week 32 of gestation until the postpartum period. The control group received standard antepartum and postpartum care. Prolactin and oxytocin levels were measured in weeks 1 and 6 postpartum. Mean prolactin increment was significantly higher in the intervention group (176.8 ± 66.6 ng/ml) than the control group (24.8 ± 39.5 ng/ml). Similarly, mean oxytocin increment was significantly higher in the intervention group (58.6 ± 31.59 pg/ml) than the control group (14.6 ± 36.06 pg/ml). Our results suggest that yoga training in the third trimester until the postpartum period increases prolactin and oxytocin levels among primipara postpartum women.

## INTRODUCTION

Breastfeeding needs to be initiated as soon as possible after delivery to stimulate the effective synthesis and secretion of breast milk. Early initiation of breastfeeding, exclusive breastfeeding for 6 months, and optimal breastfeeding for up to 2 years is essential for the optimal growth and development of infants. The World Health Organization has set a target for 2025, aiming for half of all mothers worldwide to exclusively breastfeed their newborns. However, the global rate of exclusive breastfeeding until the age of 6 months is only 38% [1], even though the level of breastfeeding initiation has increased and showed a stable growth [1,2]. In Indonesia, the percentage of exclusively breastfed infants aged 0–6 months was 80% in 2021, while in East Java province it was only 71.50%, lower than the national level. According to the literature, physical challenges, being a primiparous mother, fatigue, physiological changes, and concerns about baby care and breastfeeding were the potential factors for early cessation of breastfeeding [3–8].

Breast milk production relies on hormonal factors such as prolactin and oxytocin [9]. An imbalance in prolactin levels can impede lactation [10], whereas low oxytocin levels postpartum may hinder the milk ejection reflex, affecting milk release [11]. Reduced oxytocin levels can subsequently lower prolactin levels, leading to decreased milk synthesis and early cessation of breastfeeding [12]. These changes can adversely affect the infant’s growth, sleep, and body weight [13–16]. Establishing adequate milk production by 6 weeks postpartum is crucial for successful breastfeeding and requires effective interventions to enhance lactation [8], and increase awareness on maternal and infant health [15].

In Indonesia, health education encompasses various topics, such as the importance of exclusive breastfeeding, breastfeeding positions and techniques, postpartum nutrition, an overview of hormones involved in lactation, and dietary choices that promote breast milk production. Yoga is known for its relaxation benefits, helping to alleviate stress and tension [17]. In the postpartum period, yoga can trigger the release of endorphins, promoting feelings of comfort and relaxation [17,18], which can stimulate the release of oxytocin [12,19]. Previous studies have shown a positive correlation between oxytocin and cortisol levels in breastfeeding women experiencing stress symptoms [19].

Thus far, yoga has been used for pregnant women with beneficial outcomes, including reduced depression, stress, and anxiety [20–24], lowered incidence of preeclampsia and premature birth [25], and improvements in blood pressure, fetal heart rate [20], sleep quality [23,26], self-efficacy [27], and prenatal attachment [24,27]. In the postpartum and breastfeeding periods, yoga training has been implemented to enhance uterine involution, breast milk production, breastfeeding self-efficacy, quality of life, and maternal attachment [28–30]. Notably, research exploring the association between yoga and prolactin levels has been limited to women with multiple sclerosis, using online tele-yoga and tele-Pilates exercise interventions via platforms such as Google Meet, Zoom, and Instagram [31].

Currently, yoga classes target pregnant women, aiming to enhance relaxation, pelvic muscle flexibility, and preparation for childbirth [24]. However, postpartum and breastfeeding yoga classes have not yet been introduced in midwifery service units in Indonesia, and to the best of our knowledge, this study represents the first initiative to provide yoga exercises for postpartum mothers. The research hypothesis was that the implementation of yoga training would increase prolactin and oxytocin levels in primipara women postpartum and would improve their quality of life. Addressing these issues may mitigate future financial burdens and reduce the need for medication among mothers and infants [32,33].

The aim of this study was to evaluate the effect of yoga training on postpartum prolactin and oxytocin levels in primipara women.

## MATERIAL AND METHODS

### Study design and setting

This study used a quasi-experimental design featuring pretest-posttest comparison with a control group. The research was conducted between July and December 2022 at Adan-adan, Bendo, Gurah, and Sidorejo primary healthcare centers, which provide care for the largest numbers of primigravida mothers in Kediri Regency.

### Participants

**Figure 1:**
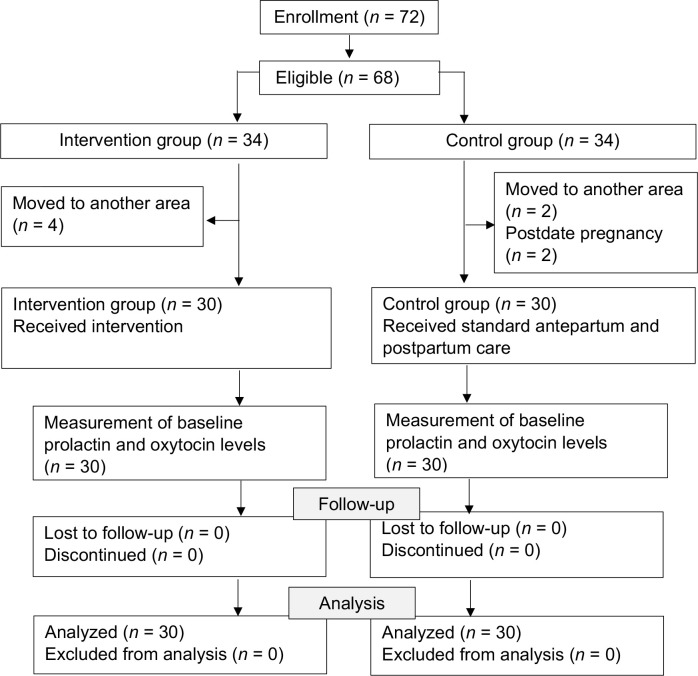
Subject selection diagram

Participants for the intervention group were selected from Adan-adan and Sidorejo primary healthcare centers, whereas participants for the control group were selected from Gurah and Bendo primary healthcare centers. The inclusion criteria were first pregnancy with gestational age > 32 weeks, age 20–35 years, willingness to participate, antenatal visits at least once in the first and once in the second trimester, mid-upper arm circumference > 23.5 cm, body mass index (BMI) 18.5–24.9, normal nipples, normal delivery, early initiation of breastfeeding, infant born on term, alive and without abnormalities. The exclusion criteria were contraindications to yoga training such as heart disease, asthma, or injury, anemia, thyroid disease, and chronic diseases. Sample size calculation was based on a 5% type I error, 80% power, a variance of 5.05 [34], and a minimum mean difference of 1.7, resulting in a minimum sample size of 28 in each group. To account for potential dropouts, 60 subjects were ultimately included in the study. The subject selection diagram is presented in Figure 1.

### Intervention

The intervention group (*n* = 30) received standard antepartum and postpartum care, health education, and eight yoga training sessions, four in the third trimester, after 32 weeks of gestation, and four in the postpartum period. The health education material provided information about breastfeeding, signs of infant satisfaction, postpartum nutrition, diets that enhance lactation, the influence of hormones on lactation, and the incorporation of exercise and yoga during lactation. Yoga sessions lasted for 60 min and included centering (10 min), pranayama (10 min), asana (30 min), and relaxation (10 min). Asana poses included Baddha Konasana, Uppavista Konasana, Dandasana, Table Pose, Adho Mukha Virasana, Bitilasana Marjariasana, Tadasana, Utkatasana, and Savasana. The relaxation phase consisted of Savasana. The yoga sessions were conducted with soft, calming background music.

The control group (*n* = 30) received standard postpartum care, which included monitoring of vital signs (blood pressure, pulse, respiration, and temperature), assessment of uterine involution, examination of lochia and bleeding, genitalia, and breasts, guidance on exclusive breastfeeding, administration of vitamin A supplements, offering contraceptive services, and addressing high-risk complications during the postpartum period [35]. Standard postpartum care was provided at four scheduled intervals based on Indonesian guidelines: the first assessment occurred at 6–48 h postpartum, followed by subsequent assessments at 3–7 days, 8–28 days, and 29–42 days postpartum. Age, parity, BMI, energy, protein, and fluid intake, anxiety levels, and social support were considered as potential confounding factors.

### Data collection tools

In conducting the research, the researchers were assisted by four midwives, four nutritionists, and four laboratory staff working at the selected healthcare centers in Kediri Regency. The intervention was carried out by two registered midwives who held yoga facilitator certificates, were certified lactation counselors, and had least 5 years of clinical experience in midwifery, along with a bachelor’s degree in midwifery education. Baseline demographic data included education level, employment status and income, obtained from maternal and child health records, identification documents, and interviews. Baseline clinical characteristics included maternal age, gestational age, infection status, the time of breastfeeding initiation, mid-upper arm circumference, BMI, energy and protein sufficiency levels, fluid intake, social support, and anxiety levels. Data were collected through interviews using structured questionnaires and measurements. Data regarding energy, protein and fluid intake were collected by nutritionists using the 2007 Nutrisurvey program. Clinical characteristics were assessed in the first week postpartum. Food consumption data were obtained via interviews using a 2 × 24-h food recall form filled out once during weekdays and once during the weekend. Social support was assessed using the Medical Outcomes Study-Social Support Survey (MOS-SSS) instrument, comprising four domains: emotional/informational support, tangible support, positive social interaction, and affectionate support. The reliability of the MOS-SSS instrument is 0.96, with a Cronbach’s alpha coefficient between 0.83 and 0.97 for each domain. The validity of the MOS-SSS instrument has a positive and significant correlation (0.69–0.82) between each domain and each other [36]. Anxiety levels were measured using the State-Trait Anxiety Inventory (STAI) [37,38], which consists of 20 statement items: 10 positive items related to feelings of safety and comfort, scored from 1 (‘very much so’) to 4 (‘not at all’), and 10 negative items related to fear, anxiety, and tension, scored from 1 (‘not at all’) to 4 (‘very much so’). The degree of anxiety is determined based on the total score for the 20 items, a score of 20–31 being classified as normal, 32–43 classified as mild anxiety, 44–55 as moderate anxiety, 56–67 as severe anxiety, and 68–80 as panic. The STAI instrument has a very good internal scale consistency, with a Cronbach’s alpha coefficient of 0.93 and an intra-class correlation coefficient of 0.8 [37]. Prolactin and oxytocin levels were measured at the Diponegoro University Iodine Deficiency Disorders Laboratory at Diponegoro National Hospital, Semarang, using ELISA [31], on week 1 postpartum as pretest and on week 6 postpartum as posttest. Blood samples were centrifuged to obtain serum and stored in a refrigerator at a temperature of 2–6 °C.

### Statistical analysis

Demographic data, as well as descriptive and inferential statistics were analyzed using SPSS 26 (IBM Corp). Descriptive statistics were used to characterize the frequencies of the study variables. Continuous variables are reported as mean ± s.d., whereas categorical variables are expressed as counts and percentages. Differences between categorical data were assessed with the chi-squared test, and those between normally distributed numerical data with independent *t*-tests. Mean prolactin and oxytocin levels before and after the intervention were compared using independent *t*-tests, and mean differences were compared using paired *t*-tests. The mean increments of prolactin and oxytocin levels between the intervention and control groups were evaluated using independent *t*-tests. *P* values of <0.05 were considered statistically significant.

## RESULTS

Based on Shapiro–Wilk normality test results, prolactin and oxytocin levels at baseline and at the end of intervention were normally distributed in both groups. Levene’s test also showed that prolactin and oxytocin levels had equal variances in both groups.

### Demographic characteristics

Baseline demographic characteristics, education levels, and employment status of the study participants are presented in Table 1. In total, 50% of women in the intervention group had a high school education, 66.7% were housewives, and 83.3% had income levels above the minimum wage. Similarly, 53.3% of women in the control group had a high school education, 53.3% were housewives, and 76.7% had income levels above the minimum wage. The results of the chi-squared test showed that there were no significant differences in education level (*P* = 0.405), employment status (*P* = 0.292), and income (*P* = 0.228) between the intervention and control groups (Table 1).

**Table 1:** Demographic characteristics in the intervention and control groups

Demographic characteristics	Intervention group (*n* = 30)		Control group (*n* = 30)		*P* value
	*n*	%	*n*	%	
**Education**					
Middle school	9	30	6	20	0.405
High school	15	50	16	53.3	
University	6	20	8	26.7	
**Employment status**					
Employee	10	33.3	14	46.7	0.292
Housewife	20	66.7	16	33.3	
**Family income**					
Lower than minimum wage	5	16.7	7	23.3	0.228
Equal to or higher than minimum wage	25	83.3	23	76.7	

*P* value from chi-squared test, significant at <0.05

### Clinical characteristics

Baseline clinical characteristics, maternal age, social support scores, anxiety levels, food and fluid intake are presented in Table 2. The majority of participants did not have an infection in the third trimester of pregnancy; however, in the postpartum period, 6.7% of women in the intervention group had an upper respiratory tract infection and 3.3% had mastitis, and 6.7% of women in the control group had mastitis. Women with mastitis received analgesic, antibiotic, and antipyretic treatment.

There were no statistically significant differences in infection status, the time of breastfeeding initiation, maternal age, social support scores, anxiety levels, as well as energy, protein, and fluid intake between the groups (Table 2).

**Table 2:** Clinical characteristics in the intervention and control groups

Clinical characteristics	Intervention group (*n* = 30)	Control group (*n* = 30)	95% CI	*t* value	*P* value
**Infection during the study, *n* (%)**					
**Upper respiratory tract infection**	2 (6.7%)	0 (0%)			0.640*
**Mastitis**	1 (3.3%)	2 (6.7%)			
**No infection**	27 (90%)	28 (93.3%)			
**Time of breastfeeding initiation, *n* (%)**					
**First 30 min**	8 (26.7%)	10 (33.3%)			0.201*
**1–4 h**	16 (53.3%)	15 (50%)			
**5 h or later**	6 (20%)	5 (16.7%)			
**Maternal age (years), mean ± s.d**.	22.97 ± 3.01	23.40 ± 2.46	0.99–1.89	0.611	0.544**
**Social support scores, mean ± s.d**.	65.90 ± 11.12	64.90 ± 8.22	0.09–10.03	1.966	0.054**
**Anxiety levels, mean ± s.d**.	27.00 ± 3.62	29.73 ± 3.85	0.74–7.46	–2.442	0.660**
**Energy sufficiency level (%), mean ± s.d**.	92 ± 4.28	90 ± 5.92	5.82–9.28	0.460	0.648**
**Protein sufficiency level (%), mean ± s.d**.	82 ± 8.62	86 ± 8.85	4.23–7.03	–0.498	0.620**
**Fluid intake (ml), mean ± s.d**.	2,250 ± 388.41	2,300 ± 427.50	161.09–261.09	0.336	0.637**

* Chi-squared test, **Independent *t*-test

### Prolactin levels

Pre- and posttest mean prolactin levels in the intervention and control groups are presented in Table 3. There were no differences in mean prolactin levels between the intervention and control groups at pretest (*P* = 0.893); however, the mean prolactin level was significantly higher in the intervention group at posttest, increasing from 209.8 ± 29.81 ng/ml to 386.6 ± 53.74 ng/ml (*P* < 0.001). In the control group, there was no difference between the mean prolactin levels at pre and post-test (*P* = 0.092). The mean increment of prolactin levels in the intervention group was significantly larger (176.8 ng/ml) than in the control group (24.8 ng/ml) (*P* < 0.001). These results suggest that yoga training may increase prolactin levels.

**Table 3:** Pre- and posttest mean prolactin levels in the intervention and control groups

Prolactin level (ng/ml), mean ± s.d.	Intervention group (*n* = 30)	Control group (*n* = 30)	95% CI	*t* value	*P* value
**Pretest**	209.8 ± 29.81	211.0 ± 36.90	16.18–18.51	0.135	0.893**
**Posttest**	386.6 ± 53.74	235.8 ± 42.00	125.86–175.72	−12.108	<0.001**
***P* value**	<0.001*	0.092*			
**Increment**	176.8 ± 66.56	24.8 ± 39.51	123.66–180.24	−10.752	<0.001**

* Paired *t*-test, **Independent *t*-test

### Oxytocin levels

Pre- and posttest mean oxytocin levels in the intervention and control groups are shown in Table 4. At pretest, there was no difference in the mean oxytocin levels between the intervention and control groups (*P* = 0.066). However, the mean oxytocin level of the intervention group was significantly higher at posttest, increasing from 189.7 ± 21.37 pg/ml to 248.3 ± 27.59 pg/ml (*P* < 0.001). There was no difference between the mean oxytocin levels at pre- and posttest in the control group (*P* = 0.493). The increment of mean oxytocin levels was significantly higher in the intervention group than the control group (*P* < 0.001). These results suggest that yoga training may increase oxytocin levels.

**Table 4:** Pre- and posttest mean oxytocin levels in the intervention and control groups

Oxytocin level (pg/ml), mean ± s.d.	Intervention group (*n* = 30)	Control group (*n* = 30)	95% CI	*t* value	*P* value
**Pretest**	189.7 ± 21.37	204.7 ± 19.88	4.39–25.73	2.287	0.066**
**Posttest**	248.3 ± 27.59	209.3 ± 33.52	23.11–54.85	−4.918	<0.001**
***P* value**	<0.001*	0.493*			
**Increment**	58.6 ± 31.59	14.6 ± 36.06	36.53–71.56	−6.175	<0.001**

* Paired *t*-test, **Independent *t*-test

## DISCUSSION

The intervention in this study was carried out in groups because it has more advantages than individual care. Previous studies found that mothers who received group care had five times greater satisfaction compared to individual care [39,40]. A study that assessed the effect of yoga on breastfeeding in mothers with babies aged 1–6 months showed an increase in breast milk production by an average of 110.97 ml [29]. Another study that involved 45 women with multiple sclerosis who did tele-yoga and tele-Pilates for 8 weeks showed an increase in prolactin levels from 20.33 ± 11.72 ng/ml to 32.62 ± 23.84 ng/ml (*P* = 0.004). In that study, the yoga and Pilates sessions included three phases, similarly to our study: warm-up (10–15 min), main poses (30–40 min), and cool-down (10–15 min), and the control group received standard care [31].

In the current study, mean prolactin levels increased significantly in the intervention group, from 209.8 ng/ml at pretest to 386.6 ng/ml at posttest. The results are in accordance with the theory that prolactin levels during the first 10 days of lactation have an average baseline of about 200 ng/ml, with a further increase to peak levels of 400 ng/ml after breastfeeding [41]. Prolactin has a major role in lactogenesis, increased prolactin secretion being essential for successful breastfeeding [42]. This increase in mean prolactin levels is in accordance with studies that showed that prolactin production can be increased through stimulation of the chest area or by avoiding stress and anxiety [10,43]. In this study, yoga practice was a mind–body–soul exercise based on breathing exercises (pranayama), physical postures (asana), and relaxation, focused on the area around the chest and breasts on the basis of complete respiration. Pranayama was done using a slow breathing technique, using the diaphragm so that the abdomen rises slowly and the chest fully expands. Maximum lung expansion increases the amount of oxygen entering the body and may have an indirect effect on key structures involved in emotional regulation, such as the hypothalamus and the limbic system [44,45]. Yoga can stimulate the production of endorphins, leading to feelings of comfort and relaxation [17,18], as well as reduce anxiety by lowering cortisol levels [17], and improve blood circulation [17,45]. Cortisol binds to special receptors located throughout the autonomic nervous system and sends positive feedback to the hypothalamic axis, stimulating the anterior pituitary to secrete prolactin [10,46] and the posterior pituitary to release oxytocin [12]. The released oxytocin stimulates the alveolar cells involved in lactation. Oxytocin release during breastfeeding also triggers the release of cortisol [19,47]. At the same time, when the posterior pituitary releases oxytocin, the hypothalamus suppresses the release of factors that inhibit prolactin secretion and stimulates the release of factors that activate prolactin secretion [10,48]. These factors trigger the anterior pituitary to discharge prolactin, thereby stimulating the alveoli cells to produce milk [49].

A study assessing the effect of yoga on oxytocin levels in a group of women with schizophrenia found that oxytocin levels were significantly higher in women who participated at yoga sessions compared to the control group (*P* = 0.01) [50]. In our study, the mean oxytocin level of the intervention group was 189.7 pg/ml at pretest and 248.3 pg/ml at posttest. These results are in accordance with previous research, which showed that the mean oxytocin level of mothers who were still breastfeeding at 7 months postpartum was 284.9 pg/ml [51]. The mean increment in oxytocin level in the intervention group (58.8 pg/ml) was higher than in the control group. These results are also in accordance with the theory that there is a correlation between oxytocin levels and lactation. Higher oxytocin levels in the early breastfeeding period are associated with increased milk production and a longer duration of lactation [12]. Breastfeeding-induced oxytocin release is associated with increased prolactin levels and decreased levels of adrenocorticotropic hormone, cortisol, and somatostatin [12,19].

The strength of this research consists in the fact that it is the first study to show the positive effect of yoga training on postpartum prolactin and oxytocin levels, which can ensure successful breastfeeding. The results of the research can be applied in midwifery service units, by adding yoga training to the standard protocols of antepartum and postpartum care. The study’s limitations include the potential for bias regarding the filling out of questionnaires to measure anxiety levels. Another limitation is the quasi-experimental design of study, as we were not able to randomize study participants in the area.

## CONCLUSION

This study found that eight sessions of yoga training started at 32 weeks of gestation were able to significantly increase prolactin and oxytocin levels in primipara women in the postpartum period. These findings suggest that yoga training can be used effectively by obstetric care providers as a complementary therapy to standard antepartum and postpartum care to increase prolactin and oxytocin levels in the postpartum period.
